# Counting the lives saved by DOTS in India: a model-based approach

**DOI:** 10.1186/s12916-017-0809-5

**Published:** 2017-03-03

**Authors:** Sandip Mandal, Vineet K. Chadha, Ramanan Laxminarayan, Nimalan Arinaminpathy

**Affiliations:** 1 Public Health Foundation of India, New Delhi, India; 2 Epidemiology and Research Division, National Tuberculosis Institute, Bangalore, India; 3 Center for Disease Dynamics, Economics, and Policy, Washington, DC USA; 4 Princeton University, Princeton, NJ USA; 5 Department of Infectious Disease Epidemiology, Faculty of Medicine, Imperial College, London, UK

**Keywords:** India, Tuberculosis, Modelling, Deaths averted

## Abstract

**Background:**

Against the backdrop of renewed efforts to control tuberculosis (TB) worldwide, there is a need for improved methods to estimate the public health impact of TB programmes. Such methods should not only address the improved outcomes amongst those receiving care but should also account for the impact of TB services on reducing transmission.

**Methods:**

Vital registration data in India are not sufficiently reliable for estimates of TB mortality. As an alternative approach, we developed a mathematical model of TB transmission dynamics and mortality, capturing the scale-up of DOTS in India, through the rollout of the Revised National TB Control Programme (RNTCP). We used available data from the literature to calculate TB mortality hazards amongst untreated TB; amongst cases treated under RNTCP; and amongst cases treated under non-RNTCP conditions. Using a Bayesian evidence synthesis framework, we combined these data with current estimates for the TB burden in India to calibrate the transmission model. We simulated the national TB epidemic in the presence and absence of the DOTS programme, measuring lives saved as the difference in TB deaths between these scenarios.

**Results:**

From 1997 to 2016, India’s RNTCP has saved 7.75 million lives (95% Bayesian credible interval 6.29–8.82 million). We estimate that 42% of this impact was due to the ‘indirect’ effects of the RNTCP in averting transmission as well as improving treatment outcomes.

**Conclusions:**

When expanding high-quality TB services, a substantial proportion of overall impact derives from preventive, as well as curative, benefits. Mathematical models, together with sufficient data, can be a helpful tool in estimating the true population impact of major disease control programmes.

**Electronic supplementary material:**

The online version of this article (doi:10.1186/s12916-017-0809-5) contains supplementary material, which is available to authorized users.

## Background

India is the highest tuberculosis (TB)-burdened country in the world, accounting for about a quarter of all incident cases and TB deaths [1]. Following a review of the erstwhile National TB Programme (NTP) in 1992, the Government of India decided to implement the Revised National TB Control Programme (RNTCP), adopting the internationally recommended Directly Observed Treatment, Short Course (DOTS) strategy [2] based on five principles: political and administrative commitment; good-quality diagnosis; uninterrupted supply of quality drugs; directly observed treatment (DOT); and systematic monitoring and accountability. Following a pilot from 1993 to 1996, the RNTCP was formally launched in 1997 and expanded in a phased manner to all the districts in the country by 2006 [3].

While DOTS remains a mainstay of TB control today, recent years have seen a shift in the ways in which DOTS programmes, such as RNTCP, are evaluated. For example, a major goal for RNTCP was to meet the primary targets of case detection of at least 70% of smear-positive incident TB cases and a cure rate of at least 85% in these cases. The TB-related Millennium Development Goals (MDGs) and the WHO’s Stop TB Strategy emphasized impact as well as programmatic implementation [4–6]: they posed targets of halving TB mortality and prevalence by 2015, compared to 1990, and reversing trends in TB incidence. More recently, the End TB strategy calls for concerted action to reduce TB deaths by 95% between 2015 and 2035 [7].

There is therefore increasing emphasis on how many TB deaths are averted (‘lives saved’) by improving TB services. The main approach to this question has been to multiply the number of patients treated under RNTCP by the percentage reduction in case fatality rates under RNTCP compared to the erstwhile NTP [8, 9]. While straightforward and transparent, a limitation of this approach is that it does not account for ‘indirect’ effects, that is, the fact that effective treatment reduces opportunities for transmission by shortening a patient’s infectious period. Lives are thus saved by averting transmission as well as by improving treatment outcomes [10, 11].

A strong vital registration system is the most direct approach for estimating lives saved. However, India’s vital registration system captures less than half of the total deaths: it has several challenges, including poorer coverage in earlier years, non-reliability of data on underlying cause of death (COD) and a high proportion of garbage coding [12–14]. These issues preclude direct measurement of TB-specific mortality rates.

Mathematical modelling offers an alternative approach, providing a systematic framework for capturing the dynamics of TB transmission and enabling estimates of program impact. Combined with Bayesian evidence synthesis methods, such models can incorporate evidence and uncertainty from a range of disparate sources, including mortality rates and epidemiological and programmatic data [15, 16].

Previous analyses by RNTCP estimated that the programme averted 1.26 million deaths from 1997 to 2006 [17], using a method similar to that described above: comparing mortality rates amongst those treated within the programme with those treated in the erstwhile NTP, which was operational since 1962 (as described, for example, in [10]). In the present work we incorporate transmission using mathematical modelling. In what follows, we give a brief overview of some essential features of RNTCP and the Indian healthcare system that are relevant to the current analysis. We then describe the model framework, the different data sources involved and the Bayesian synthesis framework. We present results for projected epidemics on a national scale, in the presence and absence of RNTCP. We show implications for the lives saved by RNTCP. Finally, we discuss implications of this work: the limitations of the model, relevance for other national settings and questions arising for future work.

## Methods

### Overview of RNTCP services

India’s RNTCP is the world’s largest national TB programme in terms of numbers of patients on TB treatment. When it was launched in 1997 to replace the erstwhile National Tuberculosis Programme (NTP), its purpose was to reform and coordinate TB services on a national level, in line with DOTS standards of TB care. In particular, improved standards of TB treatment involved enhanced adherence support amongst patients taking treatment; strengthened patient monitoring; and the use of a standard treatment regimen [2]. In turn, regarding new TB cases, in India this regimen involved four fully supervised first-line drugs (isoniazid (H), rifampicin (R), pyrazinamide (Z) and ethambutol (E)) given thrice a week for 8 weeks, followed by two partially supervised drugs (H, R) for 16 weeks. Further, to address the threat of multi-drug-resistant TB (MDR-TB), Programmatic Management of Drug-Resistant TB (PMDT) was subsequently introduced in 2007. These cases are treated with daily dosages of six drugs including second-line drugs for a minimum of 24 weeks followed by four drugs for 18 months, fully supervised over the full course of treatment.

Overall, these services had not been available in a nationally coordinated way under the erstwhile NTP and, by addressing these shortfalls, RNTCP aimed to maximize adherence and cure rates amongst patients on TB treatment. Here, we refer to these improvements collectively as ‘RNTCP services’.

However, another major feature in the Indian healthcare system is the private sector, where a substantial proportion of TB patients receive care. Vast, fragmented and unregulated, this sector provides TB care that is often substandard [18–22]. Available evidence suggests, firstly, that before RNTCP scale-up, the erstwhile NTP and the private sector had comparable standards of TB care [23, 24], and secondly, that there have not been significant improvements in the private sector since that time [20, 25]. In the present work, we therefore only distinguish RNTCP vs non-RNTCP services, taking the latter to combine both the erstwhile NTP and the private healthcare sector. We estimate lives saved by RNTCP services by comparing TB mortality against a counterfactual where TB care simply continued at the standard of the NTP and the private sector (‘non-RNTCP’), projected forward without change.

### The modelling framework

We developed a deterministic, compartmental mathematical model to capture the essential dynamics of diagnosis, treatment and transmission of TB in India, including the scale-up of RNTCP services. Key transmission and delay parameters in the model were calibrated by fitting the model to WHO estimates of incidence and prevalence in India (Table 1) [1]. Further, we used values from the literature for mortality hazards amongst cases undergoing treatment in RNTCP and amongst cases undergoing treatment elsewhere. Mortality hazards for untreated cases were obtained from studies identified in a systematic review on the natural history of untreated TB [26]. Incorporating these hazards in the transmission model, we estimated the lives saved by RNTCP between 1997 and 2016. To estimate uncertainty intervals, we embedded this process in a Bayesian framework to systematically capture contributions from uncertainty in the model inputs (mortality hazards and other parameters) as well as in the calibration targets (incidence and prevalence [27]). Further details of these components are given below.

**Table 1:** Calibration targets for the model. Estimates for incidence and proportion MDR are taken from the Global TB Report 2016, while prevalence estimates are taken from a recent pooled analysis of prevalence surveys in India, reported in [27]

Indicator	Calibration target
Incidence in 2015	217 (112–355) per 100,000 population
Prevalence in 2015	253 (195–312) per 100,000 population
Proportion MDR	3.1% (2.6–3.7) (averaged over new and retreatment cases)
Cumulative notifications to public sector (1997–2015)	19.61 million (17.65–21.57 million) (allowing for 10% error)
Out of the above, cumulative MDR notifications (2007–2015)	92,753 (83,478–102,028) (allowing for 10% error)

### Transmission model structure

**Fig. 1:**
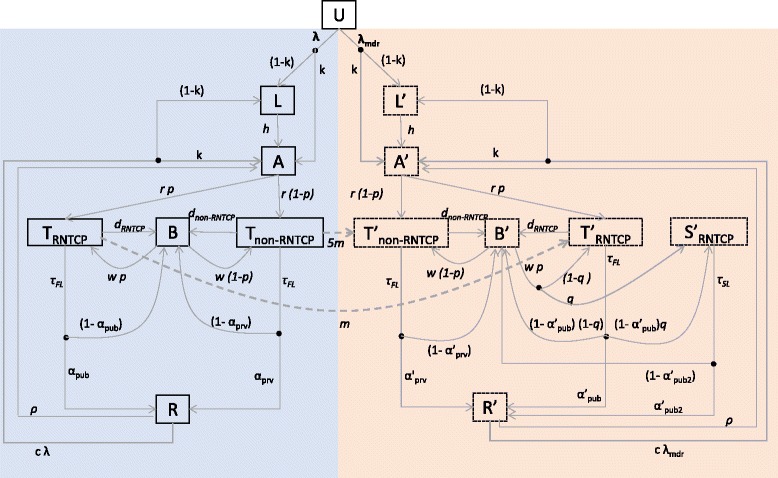
Summary of the compartmental model structure. The *left-hand side* of this figure corresponds to drug-sensitive TB, while the *right-hand side* (having compartments labelled with *dashes*) corresponds to multi-drug-resistant (*MDR*) TB. The population is divided into different compartments, representing states of disease and care seeking, with flows between compartments given by the rates shown in the diagram (see also Table 1). Concentrating on the *left-hand side* for illustration, uninfected individuals (*U*), upon acquiring infection, either enter a state of latent infection (*L*) or develop pre-treatment active disease (*A*). The rate *r* denotes the delay between the start of infectious symptoms and the first TB treatment initiation. We allow here for first-line treatment initiation either under non-RNTCP (*T*_*non-RNTCP*_) providers or under RNTCP (*T*_*RNTCP*_). From either sector a certain proportion of patients may default or fail treatment without being retained in care (*B*): these patients subsequently seek care again after a given delay. Each of these stages carries a per-capita TB mortality rate, estimated from the literature as described in the main text. Finally, individuals may be cured either through treatment or spontaneously (*R*). The *right-hand side* of this figure has slightly more complexity to account for different pathways for MDR diagnosis: these include drug resistance being recognized at the point of TB diagnosis; after non-response to first-line treatment; or not at all. The compartment S'_RNTCP_ denotes MDR-TB patients who are receiving second-line treatment in RNTCP. Further details and model equations are shown in Additional file 1. For clarity, the figure omits exogenous reinfection (which moves individuals from *R* to *L* and *I*, in the same ratios as from *U*) and relapse (which moves individuals from *R* to *I*)

Figure 1 illustrates the compartmental model structure. For simplicity, the model ignores distinctions between different forms of TB, for example, smear-positive/smear-negative/extrapulmonary TB or adult/pediatric TB. Instead, the model assumes a rate of infection (parameter *β*, to be estimated) that is essentially averaged over these different forms of TB. With less than 5% of TB cases in India being HIV-coinfected [1], we ignore the role of HIV in these dynamics.

The rate *r* (to be estimated) reflects the delay from the start of symptoms to the initiation of TB treatment under RNTCP or elsewhere: accordingly, it reflects the sum of the initial patient delay before care seeking, along with the subsequent delay before diagnosis and initiation of treatment, during which a patient may visit several different providers. The model distinguishes types of providers only at the point of treatment initiation, assuming that a proportion *p* of first-line treatment initiations are in RNTCP (the ‘T_RNTCP_’ compartment), and the remainder are elsewhere (‘T_non-RNTCP_’ compartment). As described below, we chose the time evolution of *p* to capture RNTCP scale-up. Here, the differences between RNTCP and non-RNTCP services are: (1) higher rates of treatment completion and success under RNTCP; (2) lower mortality while on treatment; (3) a lower risk of acquisition of MDR-TB from first-line treatment under RNTCP; (4) the availability of standard second-line treatment in RNTCP. For a given parameter set, we initialized the model by simulating the TB epidemic to equilibrium, in the absence of RNTCP. We then projected the model forward in time from 1997 to 2016, incorporating RNTCP scale-up from 1997 to 2007 and allowing for an annual 1.2% increase in total population size from 1997 onwards. We then assessed the model fits to the calibration data as of 2016, as described below.

Flows between compartments, including the infection process, were captured by a system of differential equations. Model parameters are listed in Table 2. Further technical details, including the governing equations, are given in Additional file 1.

**Table 2:** Parameter values used and estimated in the model. Numbers in brackets show the uncertainty ranges used in the simulations (for input parameters) or Bayesian credible intervals (for parameters being estimated)

Parameter name	Symbol	Value	Note/source
Average number of infections per drug-susceptible (DS) TB case per year	*β*	10.7 [5.8–13.6]	Estimated
Average number of infections per MDR-TB case per year	*β*_*mdr*_	2.00 [1.62–2.62]	Estimated
Per care seeking attempt, probability of seeking care in the public sector (following RNTCP scale-up)	*p*_*max*_	0.34 [0.25–0.58]	Estimated to get reported notifications from 1997–2015
Proportion of MDR-TB cases whose drug resistance is recognized at the point of TB diagnosis and who start appropriate treatment	*q*_*max*_	0.07 [0.06–0.09]	Estimated to get reported notifications from 2007–2015
Reduction in force of infection owing to previous infection	*C*	0.5	Assumed
Proportion of infections undergoing ‘rapid’ progression	*k*	0.15	Vynnycky and Fine, 1997 [42]
Rate of breakdown from remote infection to active disease	*h*	0.001 y^-1^	Horsburgh et al., 2010 [43]
Rate corresponding to the delay from the start of symptoms to the initiation of treatment (whether in public or private sector)	*r*	3.29 y^-1^ [0.83–5.70]	Estimated
Mean duration of first-line treatment	${\uptau}_{\mathrm{FL}}$	2 y^-1^	Corresponding to 6 months of treatment duration
Rate of default from non-RNTCP treatment	*d*_RNTCP_	1.06 y^-1^	Uplekar et al. 1998 [29]
Rate of default from RNTCP treatment	*d*_non-RNTCP_	0.049 y^-1^	Corresponds to 4.8% default in RNTCP (TB India, 2015 [34]) (averaged over smear-positive, smear-negative and extrapulmonary TB)
Rate of repeat care seeking after recurrence or failure	*w*	4 y^-1^	Corresponds to 3 months of delay period
Annual recurrence rate	$\uprho$	0.003 y^-1^	Corresponds to lifetime recurrence risk of 17% (Sun et al., 2013 [44])
Rate of primary MDR acquisition from patient treated under RNTCP	*m*	0.02 y^-1^	TB India, 2015 [34]
Mean duration of second-line treatment	${\uptau}_{\mathrm{SL}}$	0.5 y^-1^	Corresponding to 2 years of treatment duration
Spontaneous cure rate	$\upsigma$	0.166 y^-1^	Corresponds to 50% spontaneous cure in 3 years alongside with TB mortality (Tiemersma et al., 2011 [26])
Proportion cure of drug-susceptible (DS)-TB in RNTCP after first-line treatment	*α*_*pub*_	0.87	TB India, 2015 [34]
Proportion cure of DS-TB in non-RNTCP after first-line treatment	*α*_*prv*_	0.51	Uplekar et al., 1998 [29]
Proportion cure of MDR-TB in RNTCP after first-line treatment (excluding self-cure)	*α’*_*pub*_	0.24	TB India, 2015 [34]
Proportion cure of MDR-TB in non-RNTCP after first-line treatment	*α’*_*prv*_	0	Assumed
Proportion cure of MDR-TB with second^-^line treatment (excluding self-cure)	*α’*_*pub2*_	0.48	TB India, 2014 [45]
Per-capita mortality hazard before diagnosis	*μ*_*UTB*_	0.086 (95% CI 0.075–0.11) y^-1^	See Additional file 1
Mortality hazard during RNTCP treatment	*μ*_*RNTCP*_	0.076 (95% CI 0.069–0.095) y^-1^	See Additional file 1
Mortality hazard during non-RNTCP treatment	*μ*_*non-RNTCP*_	0.27 (95% CI 0.22–0.33) y^-1^	See Additional file 1
Mortality hazard following default and treatment failure	*μ*_*B*_	0.28 (95% CI 0.22–0.36) y^-1^	See Additional file 1

### Capturing scale-up of RNTCP services

**Fig. 2:**
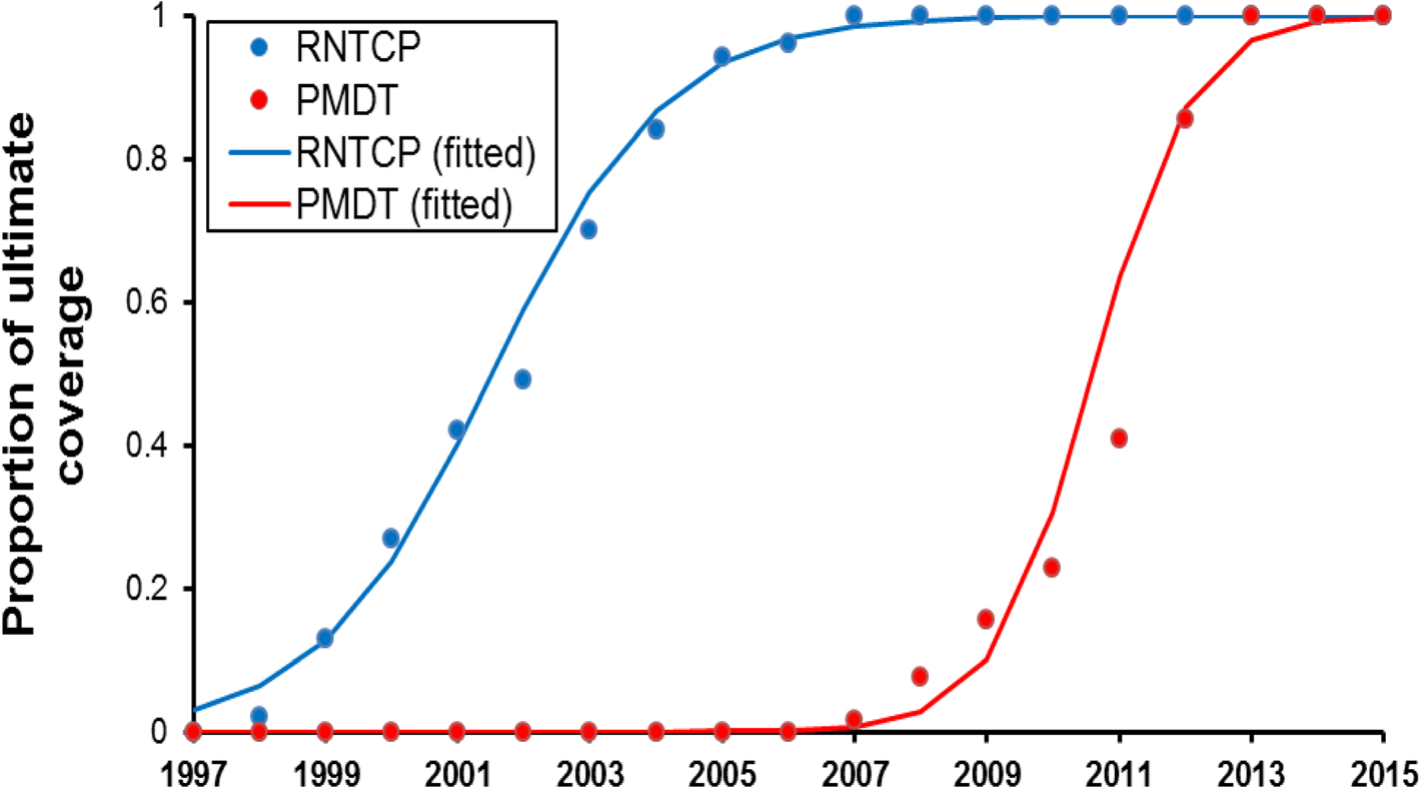
Scale-up of RNTCP services. *Blue points* show data for the proportion of geographical coverage of RNTCP [33], while *red points* show data for the proportion of geographical coverage of PMDT for MDR-TB [33]. As described in the text, these data were used to determine logistic functions capturing the timing and pace (‘steepness’) of scale-up. Resulting functions are superimposed as *blue* and *red curves*, with the following parametric forms: *F*(*t*) = 1/[1 + Exp(4 · 2 - 0 · 76* *t*)] (RNTCP scale-up), *G*(*t*) = 1/[1 + Exp(20 - 1 · 37* *t*)] (PMDT scale-up). Note that a value of 1 on the *y*-axis does not imply that the proportion of TB patients treated by RNTCP is 100%; rather, this proportion is given by *p*_max_ *F*(*t*), where *p*_max_ is a parameter to be estimated (see Methods). That is, *F*(*t*) (and similarly *G*(*t*)) simply represent the proportion of *ultimate* coverage reached, at a given time during scale-up

For the proportion of cases *p*(*t*) receiving first-line treatment through RNTCP at a given time *t*, we used programme data for RNTCP geographical coverage (Fig. 2, blue points) as a proxy for scale-up, modelled using a logistic function (Fig. 2, blue curve; see figure legend for associated coefficients). In the model, the parameter *p*_max_ (the ultimate ‘plateau’ for *p*(*t*)) was subsequently chosen to give the correct, cumulative notifications (public-sector treatment initiations) up to 2015.

Similarly, for coordinated MDR-TB services (PMDT), as a proxy for scale-up we used data for the numbers of MDR-TB cases notified to the programme (Fig. 2, red points). The red curve *G*(*t*) illustrates the logistic function used to model the pace of PMDT scale-up, with coefficients again given in the legend. In the model we took *q*(*t*) = *q*_max_ *G*(*t*), where the parameter *q*_max_ was chosen to give the correct cumulative number of notifications of MDR-TB up to 2015.

### Epidemiological data

We calibrated the model to TB incidence and prevalence. WHO estimates for TB incidence have recently been revised upwards substantially, in light of growing evidence that the true TB burden in India is greater than was previously recognized [1]. We used the updated, 2015 estimates for incidence. However, in the absence of similarly updated prevalence estimates, we drew instead from a recent analysis pooling subnational prevalence surveys at different sites in India and reported in [27], taking this estimate to represent national TB prevalence in 2015. While information on trends would be helpful, the most recent WHO estimates in incidence trends are based on further assumptions, namely that trends for incidence mirror those in the Annual Risk of TB Infection (ARTI) [1, 28]. Therefore, to avoid fitting our model to what is essentially another model, we limited the calibration only to the single time points described above, leaving it to the uncertainty estimation to capture the different trends that would meet these targets. We modelled these incidence and prevalence estimates and their accompanying 95% uncertainty intervals, using lognormal probability distributions, to incorporate these distributions in the Bayesian melding procedure described below.

### Mortality hazards

Owing to challenges with vital registration data [9, 12], we drew from the literature for different mortality hazards in the model. In particular, we populated four different types of mortality: amongst untreated TB (*μ*_UTB_), amongst those receiving treatment in RNTCP (*μ*_RNTCP_); amongst those receiving treatment outside RNTCP (*μ*_non-RNTCP_); and amongst those who have undergone failure or default (*μ*_B_).

For data on *μ*_UTB_, sources were drawn from a systematic review [26]. We analysed the survival data from these studies to estimate the overall hazard of mortality, allowing for variation between studies using exponential regression with random effects. For *μ*_RNTCP_, as discussed in the Additional file 1, we drew from programmatic data, constructing uncertainty to capture the annual variability in these data. There was less information on *μ*_non-RNTCP_ and *μ*_B_, each informed by only one source in the peer-reviewed literature [29, 30]. Accordingly, we adopted estimates from these respective sources, while allowing for broad uncertainty around these estimates.

### Estimating lives saved

For a given set of model parameters, we simulated the numbers of TB deaths in the presence and absence of RNTCP services from 1997 to 2016, estimating lives saved as the excess deaths between these two scenarios, and assuming no change in the ‘non-RNTCP’ standard of care over this period. The uncertainty in key model inputs (primarily, per-capita mortality hazards) and calibration targets (incidence, prevalence and percent MDR in 2015) gives rise to uncertainty in estimates for model parameters (*β*, *β*_MDR_, *r*, *p*_max_ and *q*_max_) and thereby in model estimates for lives saved. To drive this ‘propagation’ of uncertainty from inputs to outputs in a systematic way, we incorporated the error probability distributions described above for mortality hazards, incidence and prevalence, together with uniform priors for the model parameters, in a Bayesian posterior density (see Additional file 1 for further details). We used Bayesian melding [31] to sample from this posterior density to accumulate 250,000 samples for the model parameters. For each sample we then calculated the number of lives saved, to find an ensemble of estimates. From this ensemble we extracted the 2.5^th^, 50^th^ and 97.5^th^ percentiles, ultimately to gain point and uncertainty estimates for the lives saved by RNTCP. We refer here to these uncertainty estimates as ‘credible intervals’ (CrI), to distinguish them from the ‘confidence intervals’ arising from frequentist statistical approaches.

We also sought to distinguish the TB lives saved directly by improved programmatic conditions from the lives saved as a result of reduced opportunities for transmission: to separate such ‘direct’ and ‘indirect’ effects, we sought to estimate direct effects alone, by controlling for transmission. In particular, we calculated the force of infection ${\lambda}_{RNTCP}(t)$ acting in the simulated, ‘RNTCP’ scenario. Controlling for transmission effects requires that the same force of infection, as a function of time, should apply even in the absence of RNTCP. Accordingly, we simulated this latter scenario with the force of infection given not as a function of prevalence (as is usually the case), but imposed as the pre-determined ${\lambda}_{RNTCP}(t).$ We then calculated the lives saved owing to direct effects as the difference in TB mortality between these scenarios. Further details are provided in Additional file 1.

## Results

**Fig. 3:**
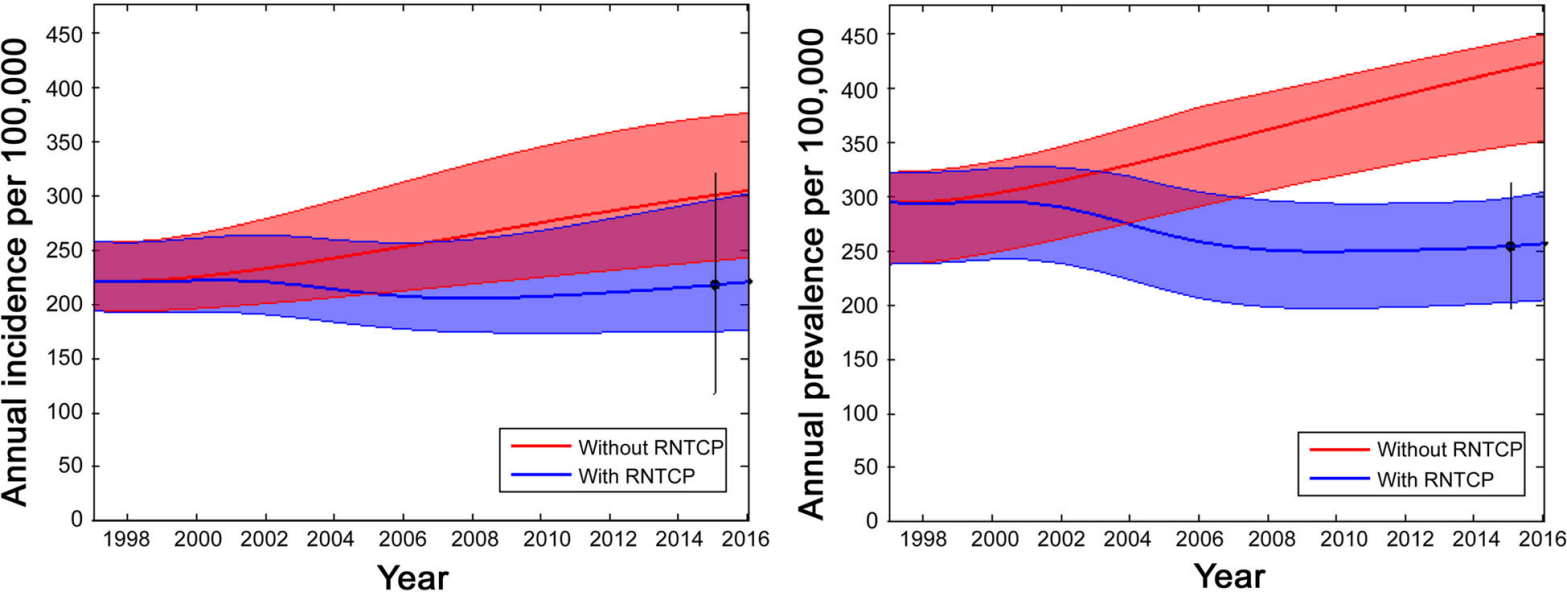
Model projections for annual TB incidence and prevalence, showing projections in the presence of RNTCP (*blue region*) and in its absence (*red region*). To construct these regions, incidence and prevalence curves were determined for each of the parameter sets in the sampled posterior distribution. From the resulting set of curves, upper and lower boundaries for the trajectories were determined using the 2.5^th^ and 97.5^th^ percentiles for incidence and prevalence at each time point. The *bold lines* represent the epidemic trajectories corresponding to the maximum posterior density (best-fitting parameter set). *Circles* and uncertainty intervals in *black* represent WHO estimates for incidence and prevalence

Table 2 gives the estimates for the model parameters. Epidemic trajectories implied by these estimates are illustrated in Fig. 3, showing projections for incidence and prevalence in the presence and absence of RNTCP. The figure also illustrates the simulation uncertainty in these trajectories arising from the uncertainty in model inputs.

**Fig. 4:**
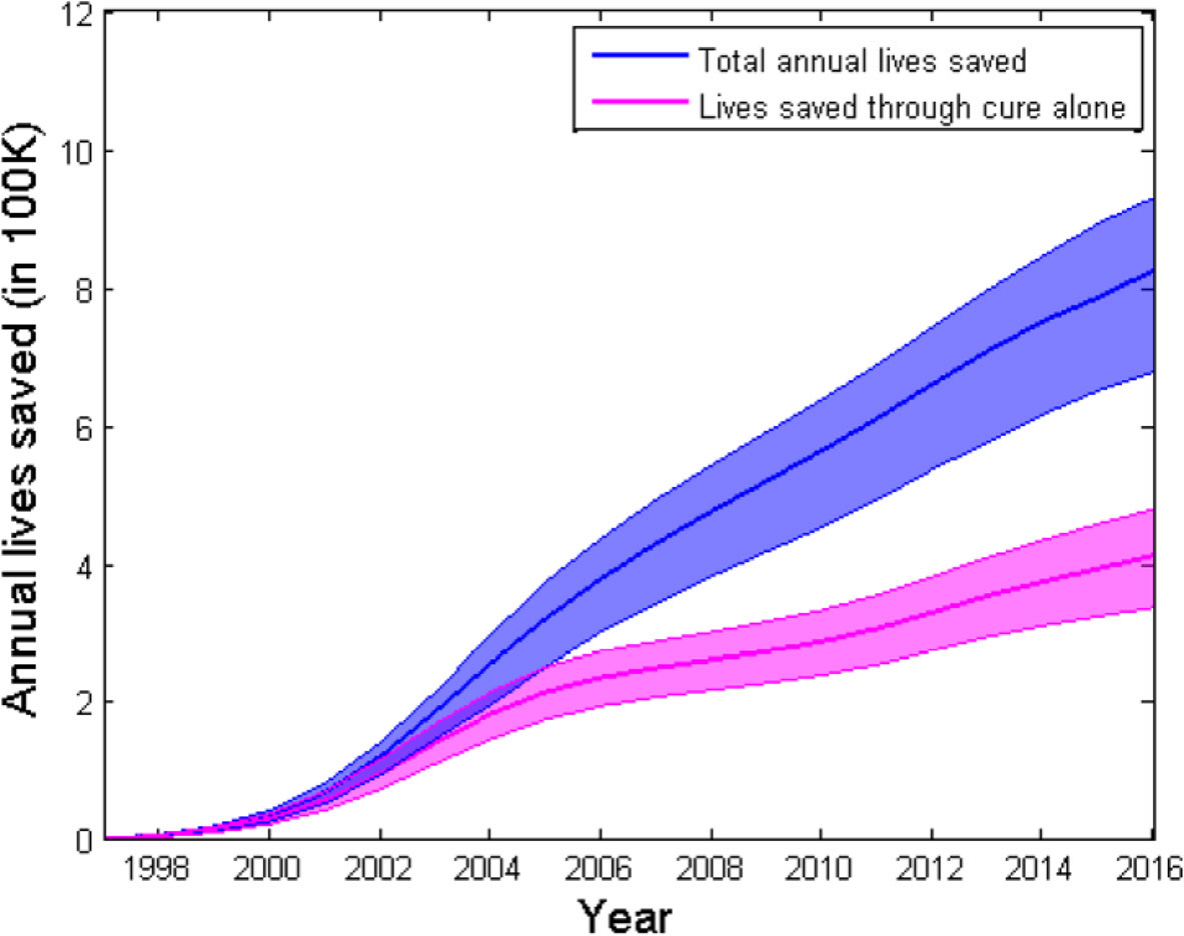
Model projections for annual lives saved by RNTCP since 1997. The *shaded region*, showing a 95% credible interval for the epidemic trajectory, is constructed as described in Fig. 3. The upper region shows overall cumulative lives saved each year, while the lower region aims to control for reducing transmission over time, to show lives saved directly through improved treatment outcomes alone. Broadly, the vertical separation between these regions can be interpreted as the lives saved through indirect effects (reducing transmission)

Additional file 1: Figure S1 shows estimates for the annual numbers of TB-related deaths in the presence and absence of RNTCP. The difference between these two scenarios gives the annual lives saved by RNTCP, presented in Fig. 4, together with a separation of direct and indirect effects (see also Table 3 for numbers supporting these plots). Overall, these results suggest that between 1997 and 2016, there were 7.75 million lives (95% CrI 6.29–8.82 million) saved by RNTCP. Of this impact, 3.28 million lives (95% CrI 2.58–4.02 million) (roughly 42%) were attributable to ‘indirect’ effects of reducing transmission, the remainder attributable to the direct programmatic effects that would have arisen in the absence of any transmission impact. Additional results (Additional file 1: Table S3) suggest that a total of 42.48 million TB patients (95% CrI 33.94–47.91 million) were cured through TB treatment over the same period.

**Table 3:** Estimated deaths averted by RNTCP from 1997–2016 *CrI* credible interval

	Direct effects	Indirect effects	Total lives saved
DS-TB lives saved	3.53 million (95% CrI* 2.86–4.11 million)	2.57 million lives (95% CrI 1.80–3.09 million)	6.25 million lives (95% CrI 4.96–7.14 million)
MDR-TB lives saved	0.71 million (95% CrI 0.61–0.79 million)	0.76 million (95% CrI 0.60–0.96 million)	1.50 million lives (95% CrI 1.22–1.74 million)
Total lives saved	4.23 million lives (95% CrI 3.52–4.89 million)	3.28 million lives (95% CrI 2.58–4.02 million)	7.75 million lives (95% CrI 6.29–8.82 million)

(*): As described in the main text, CrI denotes ‘credible intervals’.

Additional file 1: Table S3 additionally separates lives saved by drug susceptibility status, suggesting that RNTCP and PMDT together averted 1.50 million MDR-TB deaths (95% CrI 1.22–1.74 million) from 1997–2016. However, not all of this impact was due to PMDT: because of the effects of RNTCP in reducing transmission even before the initiation of PMDT in 2007, there were fewer patients on first-line treatment than might otherwise have occurred, and thus fewer opportunities for the acquisition of drug resistance during treatment. Overall this amounts to a substantial indirect effect in averting cases of MDR-TB.

## Discussion

Nationally coordinated, high-quality TB services, as embodied by the DOTS strategy, are key in improving outcomes for patients with TB [32]. A difficult but important task is to project from this patient-level perspective to understand population-level impact. Here we have applied a mathematical model of TB transmission to address this need, in the context of the world’s largest national TB programme using DOTS. Our results suggest that, from 1997 to2016, India’s RNTCP has saved 7.75 million lives with roughly 42% of this impact arising from preventive, rather than curative, benefits.

As noted earlier, previous work estimated that RNTCP averted 1.26 million TB deaths from 1997 to 2006 [17]. By comparison, our work suggests a higher total impact of 1.41 million lives (95% CrI 1.08–1.62 million) saved over this period (note that Table 3 shows lives saved from 1997 to 2016). A major reason for this difference is that previous studies did not take into consideration the indirect effects due to reduced transmission and the lives saved from MDR-TB. Moreover, we have developed a model considering several parameters that govern the disease dynamics in individuals and in the community, while earlier estimations were based solely on the difference in case fatality rates under RNTCP and non-RNTCP conditions. As another comparison, our model suggests that in 2015, TB mortality in India amounted to 58 (38–75) deaths per 100,000 population. This is roughly comparable with, although somewhat higher than, independent WHO estimates of 36 (29–45) TB deaths per 100,000 population, in 2015 [1].

While a full cost-effectiveness analysis is beyond the scope of this study, we consider drug-susceptible (DS)-TB as a rough indication (separating MDR-TB, as its disproportionate costs would otherwise obscure the cost-impact ratio amongst DS cases). With an overall expenditure of USD 2710 million from 1997–2015 excluding PMDT (see Additional file 1: Table S4) [33, 34], and an estimated 5.64 million lives saved from TB over this time, our results suggest that it has cost USD 480 per DS-TB death averted.

However, TB in India has much ground still to cover. In particular, much of TB treatment happens not under the RNCTP, but in a vast and unregulated private healthcare sector, with evidence of substandard care [18, 21, 35, 36]. Correspondingly, our results suggest that — while TB incidence in India has indeed been reduced over time — it has nonetheless settled at a new plateau (Fig. 3). In practice, this plateau reflects the limit of what could be achieved through the current public sector alone. Engagement with the private healthcare sector, as well as addressing inefficiencies in the public health system itself, will be key in addressing this substantial remainder of India’s TB burden [35, 37].

Our work could also be applied to other national contexts. For example, Indonesia and other high-burden countries in the region also face challenges of a lack of reliable vital registration data [38]. China has completed several national prevalence surveys, offering evidence for actual epidemiological trends through time in that country [39]. On the other hand, for settings such as those in South Africa [40], it is not possible to neglect HIV/TB coinfection as we have done in the present work: a more developed model will be a valuable extension for future work.

As with any modelling study, there are limitations to note. In the absence of adequate data we have neglected secular trends that may have affected TB transmission over the last two decades, such as growing urbanization; an increasing prevalence of comorbidities, including diabetes; and changes in socioeconomic and living conditions. We have also used WHO incidence estimates, themselves derived from certain assumptions. Nonetheless, our findings should be seen as a demonstration of principle that could be refined with improved TB burden estimates from India. More robust data on the trends of TB burden over time (for example, through a series of prevalence surveys) could help to refine the model estimates. Moreover, in modelling the TB epidemic at country level, we have neglected subnational differences such as urban vs rural TB, as well as the unique burden of MDR-TB in locations such as Mumbai. Further work could aim to extend this analysis to these settings.

We have also made several model simplifications, for example, assuming a fixed delay before initiating treatment (even if this delay is estimated). If RNTCP scale-up meant that patients were diagnosed earlier, or indeed that TB patients would more readily seek care at newly available facilities, this would have reduced delays to diagnosis. An assumption of constant *r* would thus be conservative with respect to RNTCP impact. In modelling the absence of the RNTCP under the counterfactual scenario, we have also assumed that the standard of TB care in non-RNTCP services has remained roughly constant over time [20, 25]. Nonetheless, if non-RNTCP practices have in fact improved in India, this may reduce the overall lives saved by RNTCP. For simplicity we have also assumed that there is no difference in long-term outcomes between patients completing RNTCP and non-RNCTP treatment. With no adherence support in the private sector, however, patients stopping treatment early may face increased risks of relapse [41], with attendant risks of TB mortality. Once again, ignoring this would tend to be conservative with respect to RNTCP impact. We have also neglected age structure and the differential mortality that is likely to apply amongst pediatric cases of TB [42], given the particular challenges in diagnosing these cases. Another limitation is that we have not segregated the lives saved amongst smear-positive and smear-negative cases. Given the differential case fatality rates as observed amongst untreated smear-positive and smear-negative cases and that more emphasis has been given to smear-positive case detection (cases having a higher mortality hazard [26]), lives saved are likely to be underestimates. Finally, in the absence of reliable vital registration data, we have had to construct mortality estimates from the available literature. Ideally these estimates could be validated against in-country mortality data; while these data are currently not sufficiently robust [12], future TB mortality studies — both nationally and on the subnational level — would be invaluable in informing and refining these estimates.

## Conclusions

While there remains much ground to be covered in managing India’s TB epidemic, it is also valuable to note that the RNTCP has had a substantial impact over the past two decades. In the present work, we show that over 40% of the overall impact of DOTS in India could be attributed to reduced transmission. Complex though this impact may be, mathematical modelling can offer a helpful tool for understanding these effects, both in India and for TB control programmes elsewhere.

## Additional file

Additional file 1: Model specification and additional technical details. (DOCX 165 kb)

## Abbreviations

DOTS: Directly Observed Treatment Short Course
MDR-TB: Multi-drug-resistant tuberculosis
PMDT: Programmatic Management of Drug-Resistant Tuberculosis
RNTCP: Revised National Tuberculosis Control Programme
TB: Tuberculosis
WHO: World Health Organization
