# Supplementary material for: Counting the lives saved by DOTS in India: a model-based approach
Source: BMC Med. 2017 Mar 3;15:47. doi: 10.1186/s12916-017-0809-5 (PMC5335816; doi:10.1186/s12916-017-0809-5)
Supplement: Additional file 1: — Model specification and additional technical details. (DOCX 165 kb) [file 12916_2017_809_MOESM1_ESM.docx]

**Additional file 1**

**Counting the lives saved by DOTS-based treatment in India: a model-based approach**

Sandip Mandal^1^, Vineet K Chadha^2^, Ramanan Laxminarayan^1,3,4^, Nimalan Arinaminpathy^1,5^

^1^Public Health Foundation of India, New Delhi, India; ^2^Epidemiology and Research Division, National Tuberculosis Institute, Bangalore, India. ^3^Center for Disease Dynamics, Economics, and Policy, Washington, DC, USA; ^4^Princeton University, Princeton, NJ, USA; ^5^Department of Infectious Disease Epidemiology, Faculty of Medicine, Imperial College, London, UK

**1. Transmission model**

The model is designed to capture two essential features of RNTCP: lower mortality of patients while on treatment, and higher rates of cure than amongst patients receiving treatment elsewhere. As described in the main text, *p* is the proportion of first-line treatment initiations that are conducted by RNTCP. For MDR-TB, testing for drug resistance is a long and costly process that is currently prioritized by the programme amongst cases with treatment history. Accordingly, we assume that there is essentially no drug sensitivity testing in the private sector, and that – within RNTCP – a proportion *q* of MDR cases having failed first-line treatment are recognized as being drug-resistant, and are thus initiated on second-line treatment. The time-evolution of *p* represents RNTCP scale-up, while that of *q* represents the subsequent expansion of PMDT (Fig. 2, Main text).

Table S1 below shows the definitions for the state variables in the model, while Table 1 in the main text lists the parameters involved. The model then has the following governing equations:

 (1)
 (2)

 (3)

 (4)

 (5)

 (6)

 (7)

 (8)

 (9)

 (10)

 (11)

 (12)

 (13)

 (14)

With,

$\lambda=\beta(A+T_{RNTCP}+T_{non-RNTCP}+B) /N$ (15)

$\lambda_{mdr}=\beta_{mdr} (A'+T'_{RNTCP}+T'_{non-RNTCP}+ B') /N$ (16)

For a given parameter set, the simulation is conducted in two steps: first we assume a steady annual incidence for the pre-RNTCP era, thus simulating the model to equilibrium with *p* = 0, *q* = 0. Then, from 1997 onwards, we simulate increases in *p* and *q* according to the logistic curves illustrated in Fig.2. We also allow for an annual population growth of 1.2% from1997 onwards (choosing a birth rate $b=\mu+0.01$in the above equations). Simulating further forward in time, this procedure gives projections for incidence and prevalence in 2015. These are compared against corresponding WHO estimates, as described in the section on Bayesian melding below.

**2. Model inputs**

Estimating mortality rates

We aimed to derive point and uncertainty estimates for each of the mortality rates involved in the model. As these rates were drawn from different data sources (a systematic review, programmatic data, published data and a cohort study), in each instance we tailored the uncertainty estimation to the specific type of data involved. In general, we modeled hazards as gamma distributions, and proportions as beta distributions. Details are as follows:

*Untreated TB, μ_UTB_*

Teimersma et al [1] presented a systematic review of longitudinal studies to assess TB mortality rates in the pre-chemotherapy era. In the present work, to capture TB mortality prior to diagnosis and treatment, the model requires estimates for the ‘per-capita mortality rate’, or the hazard of mortality over time. We therefore retrieved the survival data from the sources cited in [1] (see table S2), and fitted this data using an exponential regression model with random effects.

There is separate data for smear-negative and smear-positive patients: estimating the mortality hazards separately for these groups, we then simulated a mean mortality hazard as a population-weighted average, with weights given by the estimated 63% of TB cases in India that are smear-positive [2]. This yielded an overall mortality hazard of 0·086 (95% C.I. 0·075, 0·11) per year. Finally, implementing this uncertainty in the Bayesian process requires a parametric form for the uncertainty in *μ*_UTB._ We modeled this uncertainty with a Gamma distribution, using least-squares estimation to find shape and scale parameters yielding the percentiles stated above (finding values of 51·1, 0·0017 respectively).

*Mortality during RNTCP treatment, μ*_RNTCP_

We drew from RNTCP reports on treatment outcomes over the last ten years, finding the mean (*m*) and standard error (*se*) for the case fatality rate. We then estimated uncertainty in the case fatality rate as *m* +/– 1·96 *se,* to yield estimates of 7·6% (95% C.I. 6·7 – 8.8%). For incorporation into the model, it is required to translate these estimates into a probability distribution for the annual mortality hazard.

To do so, we note that the case fatality rate is defined with respect to treatment completion as an endpoint. In the model, this is accounted for in terms of ‘competing hazards’, meaning that the proportion of patients dying before completing treatment is

$$CFR=\frac{\mu_{RNTCP}}{\left( \mu_{RNTCP}+\frac{1}{d} \right)} ,$$

where *d* is the duration of treatment in years. Rearranging this, and taking *d* = 0·5 in the case of first-line treatment, we obtain for the mortality hazard:

$\mu_{RNTCP}=2CFR/(1-CFR)$ (15)

We first fitted a beta distribution to capture the variation in the case fatality rate given above; using 10,000 values from this distribution, we then simulated a distribution for *μ*_RNTCP_ using expression (15) above. Finally, we fitted a gamma distribution to the resulting ensemble of hazard rates, ultimately finding shape- and scale-parameters of 116 and 0·0006 respectively, for the distribution of *μ*_RNTCP_.

*Mortality during non-RNTCP treatment, μ_non-_*_RNTCP_

Despite broad evidence of poor-quality TB care outside RNTCP in India [3, 4], there is considerable uncertainty on quantitative treatment outcomes. We followed previous work [2] that estimated 24% mortality while on non-RNTCP treatment. To allow for broad uncertainty around this figure, however, we allowed for a 25% error: that is, a range from 18% to 30%.

Once again, for the model it is required to translate these proportions to mortality hazards. To do so we modeled CFR as a *Beta*(12·6, 39·1) distribution, capturing the uncertainty estimates given above. Sampling 10,000 times from this distribution, and simulating corresponding values of the mortality hazard using (15) above, we finally modeled the mortality hazard as a *Gamma*(47, 0·006) distribution.

*μ_B_, mortality following default and treatment failure*

We drew from a study in India [5], containing outcome data on patients after 2·5 years of initiating treatment. This study reported that, of 56 patients who had defaulted and failed treatment, and for whom outcome data were available, 28 had died after 2·5 years. Using the usual normal approximation for confidence intervals on binomial proportions, we obtained an estimate of 50% (36·9% - 63·1%) for the proportion of defaulters and failures dying over 2·5 years: we captured this uncertainty using a *Beta*(27·3, 27·3) distribution. This proportion is related to the mortality hazard *μ_B_* in the following way:

$CFR=exp(-2\cdot5\mu_{B})$, i.e. $\mu_{B}={-log(p)}/{2\cdot5}$ (16)

As above, sampling 10,000 times from the CFR distribution, and finding corresponding values for *μ_B_* using (16), we then modelled *μ_B_* as a *Gamma*(26·7, 0·011) distribution.

**3. Propagating uncertainty from model inputs and data to lives saved**

The model used here is a deterministic framework: however, it is subject to some uncertainty in the assumed parameters, a key example being the mortality hazards involved. Moreover, although model parameters are calibrated to yield WHO estimates for TB incidence and prevalence, these estimates are themselves subject to uncertainty. Each of these sources thus adds to the overall uncertainty in the model estimates for lives saved. We require a systematic method for ‘propagating’ this uncertainty from model inputs and calibration targets, to model outputs (e.g. lives saved).

For this method we draw from Raftery et al [6], a technique used, for example, by UNAIDS for HIV burden estimates [7]. We denote as $\theta$ the set of parameters subject to variation, namely: the rate of infection $\beta$; the corresponding rate for MDR-TB $\beta_{MDR}$; the mean rate of initial careseeking *r*; the proportion of cases seeking care in the public sector $p$; and the mortality hazards, $\mu_{TB}, \mu_{RNTCP}, \mu_{non-RNTCP}, \mu_{B}$. For a given set of parameters $\theta$, we define the posterior density $\pi\left( \theta\right)$ as:

$$\pi\left( \theta\right)=L\left( D | \theta\right)q\left( \theta\right),$$

where *L* is the likelihood of the data *D* given the parameters $\theta$, and *q* is the prior distribution for $\theta$. We describe both of these in turn.

*Likelihood*

Calibration targets constituting *D* are: incidence in 2015, prevalence in 2015, the percent MDR in 2015, and notifications. We modelled incidence and prevalence using log-normal distributions, with density $LN_{Inc}(.)$ and $LN_{prev}(.)$ respectively. We chose the distribution parameters to minimise the sum of squares between 2.5^th^, 50^th^ and 97.5^th^ percentiles of the distribution, and the lower, point and upper parameter estimates, respectively. Similarly, we used a beta distribution with density $B_{MDR}(.)$ to model the proportion of TB cases that are multi-drug resistant. For notifications, we allowed a nominal uncertainty of +/- 10% in this data, again modelling this using a log-normal distribution, with density $LN_{notif}(.)$.

Overall then, if parameter set $\theta$ yields an incidence $Inc(\theta)$, and similary for the other calibration targets, we calculated the likelihood as

$$L\left( D | \theta\right)=LN_{inc}\left[ Inc(\theta) \right] . LN_{prev}\left[ Prev(\theta) \right] . B_{MDR}\left[ p_{MDR}(\theta) \right] .LN_{notif}\left[ Notifs(\theta) \right]$$

In practice we adopted the log-likelihood, thus computing the sum of the logarithm of the right-hand-side densities above.

*Priors*

The role of the priors $q\left( \theta\right)$ is to incorporate any evidence, independent of the model, for the parameters subject to variation. We adopted uniform (uninformative) priors for all the model parameters with the exception of the mortality hazards, $\mu_{TB}, \mu_{RNTCP}, \mu_{non-RNTCP}, \mu_{B}$. For each of these we aimed to capture the point and uncertainty estimates shown in Table 2 in the main text, modelling each mortality hazard with a log-normal distribution. Once again, we chose the distribution parameters to minimise the sum of squares between the relevant percentiles and the corresponding estimates. Thus, defining $LM_{TB}$ as the probability density for $\mu_{TB}$ (and likewise for the remaining parameters), we calculated the priors as

$q\left( \theta\right)=LM_{TB}\left( \mu_{TB} \right) . LM_{RNTCP}\left( \mu_{RNTCP} \right). LM_{non-RNTCP}\left( \mu_{non-RNTCP} \right) . LM_{B}\left( \mu_{B} \right). u$,

where *u* is the (fixed) contribution of the uniform priors. As above, for practical purposes we computed $\log\left[ q\left( \theta\right) \right].$

*Constructing uncertainty*

We use the Sampling Importance Resampling algorithm [8] to sample from the posterior density. In particular:

1. Generate an initial ‘pool’ of 100,000 independent parameter sets $\theta^{\left( 1 \right)}, \theta^{\left( 2 \right)}, \theta^{\left( 3 \right)},\ldots$
2. For each parameter set $\theta^{\left( i \right)}$, calculate the corresponding posterior density, $\pi^{\left( i \right)}=\pi\left( \theta^{\left( i \right)} \right)$
3. Compute the sampling importance weight $w_{i}$ of parameter set *i* as $w_{i}=\pi^{\left( i \right)}/\sum_{i} \pi^{\left( i \right)}$
4. Resample 200,000 parameter sets from the initial pool, with replacement, and using sampling weights $w_{i}$, to yield a new ensemble of parameter sets, $\Theta^{\left( 1 \right)}, \Theta^{\left( 2 \right)}, \Theta^{\left( 3 \right)}, \ldots$

We then construct estimates for lives saved in the following way: for a given set of parameter values $\Theta^{\left( i \right)}$, we simulate the model defined by equations (1 – 14) to find TB mortality. Here we refer to the mapping from $\Theta^{\left( i \right)}$ to TB deaths as $M(\Theta^{\left( i \right)}|u)$, with indicator *u* where *u* = 0 signifies the counterfactual scenario (absence of RNCTP) and *u* = 1 signifies the basecase. (In the equations (1 – 14), the counterfactual is implemented simply by fixing *p*_max_ = *f*_max_ = 0). For a parameter set $\Theta^{\left( i \right)}$, we then calculate lives saved as

$$\delta_{i}=M\left( \Theta^{\left( i \right)} | u=0 \right)-M\left( \Theta^{\left( i \right)} | u=1 \right)$$

Evaluating $\delta_{i}$ for all *i,* we obtain an ensemble of values for lives saved, from which we take the 2.5^th^, 50^th^ and 97.5^th^ percentiles, as uncertainty estimates.

Next, to calculate direct effects, as explained in the main text it is necessary to control for transmission effects: that is, given the transmission dynamics in the presence of RNTCP, we wish to construct a counterfactual with the absence of RNTCP, but nonetheless with the same transmission dynamics in effect.

Here we note that, in an autonomous system such as *M*, transmission dynamics are captured by the force-of-infection term $\lambda(t)$ specified in equations (15, 16): that is, $\lambda$ is evaluated as a function of the state variables and model parameters alone. By contrast, we now define a new mapping $F(\Theta^{\left( i \right)}|\Lambda\left( t \right))$*,* which, like *M*, translates a parameter set $\Theta^{\left( i \right)}$ to TB deaths. However, while governed by equations (1 – 14), the force-of-infection for *F* is now a pre-specified function of time $\Lambda(t)$, rather than being determined by equations (15, 16). Here, we identify $\Lambda(t)$ simply as the force of infection $\lambda_{u=1}\left( t \right)$ that arises from calculating the basecase $M(\Theta^{\left( i \right)}|u=1)$. Overall then, we calculate the lives saved due to direct effects alone as:

$$\delta_{i}^{(direct)}=F\left( \Theta^{\left( i \right)}|\Lambda\left( t \right)=\lambda_{u=1}(t) \right)-M(\Theta^{\left( i \right)}|u=1)$$

We then calculate the uncertainty for $\delta_{i}^{\left( direct \right)}$ in the same way as described above, for $\delta_{i}$.

**4. Additional model projections**

**Figure S1. Model projections for annual TB-related deaths.** Green and red regions show simulated projections of TB-related deaths in the presence and absence of RNTCP respectively. The blue region shows projection of TB-related deaths due to direct effects only. The upper and lower boundaries of the trajectories were determined using the 2.5^th^ and 97.5^th^ percentiles for simulated TB deaths at each time point. The bold lines represent deaths corresponding to the maximum posterior density.

**Figure S2. Model projections for population in India.** Yellow region shows projection of population from the model, allowing 1.2% annual population growth from 1997 onwards. Red dots show data for population growth as reported by WHO.

**Table S1: Definitions of state variables in the model.** To represent drug susceptibility status, states relating to MDR-TB are denoted with a ‘dash’ (e.g. $A^{'}$ for those having active disease prior to their first TB diagnosis), and drug-susceptible states are denoted without (e.g. *A*). All state variables denote numbers in the population.

| **Symbol** | **State variable** |
| --- | --- |
| *U* | Uninfected |
| *L* | Latent infection |
| *A* | Active disease, prior to first TB diagnosis |
| *B* | Active disease, between careseeking episodes |
| *T*_RNTCP_ | Undergoing first-line TB treatment under RNTCP |
| *T*_non-RNTCP_ | Undergoing first-line TB treatment outside RNTCP (with private sector, or erstwhile NTP) |
| *R* | Recovered and non-infectious |
| *S’*_RNTCP_ | Undergoing second-line treatment under RNTCP (MDR-TB cases only) |

**Table S2**: **Survival rates for smear-positive and smear-negative pulmonary tuberculosis.**

| **Study** | **5-year survival (95% CI)** | **10-year survival (95% CI)** |
| --- | --- | --- |
| **Smear-positive tuberculosis** |  |  |
| Sinding-Larsen [9] | 57% (54%-60%) | 47% (44%-50%) |
| Trail & Stockman [10] | 50% (48%-52%) | 34% (32%-36%) |
| Backer [11] | 35% (33%-37%) | 21% (19%-23%) |
| Fürth [12], re-analyzing data collected by Krebs [13] | 30% (27%-33%) | 19% (17%-22%) |
| Magnusson [14] | 37% (33%-43%) | 27% (23%-32%) |
| Braeuning & Neisen [15] | 25% (22%-29%) | 18% (15%-21%) |
| Griep; smear-positive [16] | 51% (48%-54%) | 34% (31%-37%) |
| Baart de la Faille [17]; smear-positive | 38% (34%-42%) | 29% (25%-33%) |
| Buhl & Nyboe [18] | 45% (39%-51%) | 34% (29%-40%) |
| Berg [19] | 42% (40%-44%) | 29% (27%-31%) |
| Thompson [20]; only smear-positive | 27% (23%-32%) | 14% (11%-18%) |
| Lindhardt [21]; only smear-positive | 43% (42%-44%) | - |
| Hartley [22] | 58% (56%-60%) | - |
| Rutledge & Crouch [23] | 39% (35%-43%) | - |
| Münchbach [24] | 50% (48%-52%) | - |
| Tattersall [25]; smear-positive | - | 23% (21%-26%) |
| **Smear-negative tuberculosis** |  |  |
| Fürth [12] re-analyzing data collected by Krebs [13] | 88% (85%-91%) | 78% (74%-82%) |
| Magnusson [14] | 92% (89%-94%) | 85% (81%-88%) |
| Baart de la Faille [17]; smear-negative | 85% (82%-88%) | 75% (71%-78%) |
| Rutledge & Crouch [23] | 86% (80%-91%) | - |

**Table S3: Estimated lives saved, cases averted and number cured by drug susceptibility status from 1997-2015**. As described in the main text, ‘CrI’ denotes Bayesian Credible Intervals.

|  | **Drug susceptible TB** | | **MDR-TB** | **Drug susceptible + MDR-TB** |
| --- | --- | --- | --- | --- |
| Estimated lives saved | | 6.25 million lives (95% CrI 4·96–7·14 million) | 1·50 million lives (95% CrI 1·22–1·74 million) | 7.75 million lives (95% CrI 6.29–8·82 million) |
| Estimated cases averted | | 9·8 million (95% CrI 6·26–11·61 million) | 0·93 million (95% CrI 0·64–1·60 million) | 10·67 million (95% CrI 7.73–12.57 million) |
| Estimated cured | | 40.72 million (95% CrI 32.26–46.20 million) | 1.71 million (95% CrI 1.46–1.91 million) | 42.48 million (95% CrI 33.94–47.91 million) |

**Table S4: Expenditure on TB Control**

| **Year** | **RNTCP expenditure**  **(in million INR)** | **RNTCP expenditure**  **(in million USD)** | **Other health sector cost**  **(in million USD)** | **Total**  **(in million USD)** | **Reference** |
| --- | --- | --- | --- | --- | --- |
| 1997-2006 |  | 299 | 469 | 768 | Goodchild, Sahu et al, 2011 [26] |
| 2007-2008 | 2621·2 | 756* | 1186** | 1942 | TB India 2013, RNTCP Financial performance during 11^th^ five-year plan [27] |
| 2008-2009 | 2799·0 |  |  |  |  |
| 2009-2010 | 3120·2 |  |  |  |  |
| 2010-2011 | 3499·5 |  |  |  |  |
| 2011-2012 | 3843·4 |  |  |  |  |
| 2012-2013 | 6627·1 |  |  |  | TB India 2015, Financial performance of RNTCP in 12^th^ five-year plan [28] |
| 2013-2014 | 6670·2 |  |  |  |  |
| 2014-2015 | 8600·0 |  |  |  |  |
| Total | 37780·6 |  |  | 2710 |  |

* Considering average conversion rate: 1 USD= 50 INR

** ‘Other’ health sector costs include costs at the sub-national level, for example hospitalisation and clinic visits, as described in Goodchild, Sahu et al, 2011 [26]· These data are only available up to 2006· In the table, we extrapolate from 2007 onwards by assuming that the ratio of these costs to RNTCP expenditure remains constant through time·

**References**

1. Tiemersma EW, van der Werf MJ, Borgdorff MW, Williams BG, Nagelkerke NJD. Natural History of Tuberculosis: Duration and Fatality of Untreated Pulmonary Tuberculosis in HIV Negative Patients: A Systematic Review. PLoS ONE. 2011; 6(4): e17601. doi:10.1371/journal.pone.0017601.
2. Pandey S, Chadha V, Laxminarayan R, Arinaminpathy N, “Estimating tuberculosis incidence from primary survey data: a mathematical modelling approach” Int J Tuberc Lung Dis (in press).
3. Uplekar, M, Juvekar S, Morankar S, Rangan S, Nunn P. Tuberculosis patients and practitioners in private clinics in India. Int J Tuberc Lung Dis. 1998; **2**(4):324-329.
4. Satyanarayana S, Nair SA, Chadha SS, et al. From Where Are Tuberculosis Patients Accessing Treatment in India? Results from a Cross-Sectional Community Based Survey of 30 Districts. Pai M, ed. PLoS ONE. 2011; 6(9):e24160. doi:10.1371/journal.pone.0024160.
5. Vijay S, Balasangameswara VH, Jagannatha PS, Saroja VN, Kumar P. Treatment outcome and two & half years follow-up status of new smear positive patients treated under RNCTP. Indian J Tuberc. 2004; **51**:199–208.
6. Poole D, Raftery AE. Inference for deterministic simulation models: The Bayesian melding approach. J Am Statist Ass. 2000; 95(452):1244-1255.
7. Alkema L, Raftery AE, Clark SJ. Probabilistic projections of HIV prevalence using Bayesian Melding. Ann Appl Stat. 2007; 1(1): 229-248.
8. Rubin D. Using the SIR algorithm to simulate posterior distributions. Bayesian Statistics 3 (J. M. Bernardo, M. H. DeGroot, D. V. Lindley and A. F. M. Smith, eds.) 1988; 395–402. Clarendon Press, Oxford, U.K.
9. Sinding-Larsen CMF. On the collapse treatment of pulmonary tuberculosis. A clinical adaption and follow-up examination of the material from Vejlefjord Sanatorium, 1906-1932. Acta Med Scand*.* 1937; **91**:39–132.
10. Trail RR, Stockman GD. A report upon the experience of the patients of the King Edward VII Sanatorium, Midhurst, with particular reference to their mortality after treatment. 1931. Pulmonary Tuberculosis: Sept.
11. Backer JE. Oslo: Norske Videnskaps-Akademi; 1937· Dodeligheten blandt lungetuberkulose· En statistik undersokelse vedkommende patienter anmeldt til Oslo Helserad i 1911 – 1930 op patienter behandlet pa Vardasen Sanatorium i 1923–1934.
12. Fürth E. Zur Frage der Lebensdauer bei Aktiver Tuberkulose· Beitr Klin Tuberk*.* 1931; **76**:573–587.
13. Krebs W· Die Fälle von Lungentuberkulose in der aargauischen Heilstätte Barmelweid aus den Jahren 1912–1927. Beitr Klin Tuberk. 1930; **74**:345–379.
14. Magnusson S· Über den Verlauf und die Letalität der Tuberkulose in den verschiedenen Altersperioden. Acta Tuberc Scand. 1938;**12**: 201–263·
15. Braeuning H, Neissen A. Prognose der offenen Tuberkulose, Technik der Prognose-stellung und Rentabilität des Heilverfahrens. Zeitschr Tuberk. 1936; **75**:305–323.
16. Griep WA. [Thesis] Amsterdam: University of Amsterdam; 1939. De prognose van de open long tuberculose.
17. Baart de la Faille RL. [Thesis] Utrecht: University of Utrecht; 1939. Onderzoek naar de resultaten der tuberculose be handeling in het sanatorium “Berg en Bosch”.
18. Buhl K, Nyboe J. Epidemiological basis of tuberculosis eradication. 9. Changes in the mortality of Danish tuberculosis patients since 1925. Bull World Health Organ. 1967; **37**(6):907–925.
19. Berg G. The prognosis of open pulmonaty tuberculosis. A clinical-statistical study. Acta Tuberc Scand suppl. 1939; IV:1–206.
20. Thompson BC. Survival rates in pulmonary tuberculosis. Br Med J. 1943; **2**: 721.
21. Lindhardt M. Copenhagen: Ejnaar Munksgaard; 1939. The statistics of pulmonary tuberculosis in Denmark 1925-1934. A statistical investigation on the occurrence of pulmonary tuberculosis in the period 1925-1934, worked out on the basis of the Danish National Health Service file of notified cases and of deaths.
22. Hartley PHS, Wingfield RC, Burrows VA. Frimley, UK: Brompton Hospital Sanatorium; 1935. The expectation of survival in pulmonary tuberculosis. Brompton Hospital Reports IV.
23. Rutledge JA, Crouch JB. The ultimate results in 1654 cases of tuberculosis treated at the modern Woodmen of America sanatorium. Am Rev Tuberc. 1919; **2**:755-763.
24. Münchbach W. Das Schicksal des lungentuberkulösen Erwachsenen. Ergibnisse der Heilstattenbehandlung von annähernd 10000 Männern und Frauen. Tuberkulose-Bibliothek. 1933; **49**:64.
25. Tattersall WH. The survival of sputum-positive consumptives. A study of 1192 cases in a county borough between 1914 and 1940. Tubercle. 1947; **28**:85-96.
26. Goodchild M, Sahu S, Wares F, Dewan P, Shukla RS, Chauhan LS, Floyd K. A cost-benefit analysis of scaling up tuberculosis control in India. Int J Tuberc Lung Dis. 2011; **15**(3): 358-362.
27. Central TB Division, Directorate General Health Services, Ministry of Health and Family Welfare, Government of India- Revised National TB Control Programme TB India 2013. Available at http://www.tbcindia.nic.in. Accessed 2016 August 30.
28. Central TB Division, Directorate General Health Services, Ministry of Health and Family Welfare, Government of India- Revised National TB Control Programme. TB India 2015. Available at http://www.tbcindia.nic.in. Accessed 2016 August 30.
